# Innovative technique for pulmonary artery control in complex left upper lobectomy: Enhancing safety in minimally invasive resections

**DOI:** 10.1016/j.xjtc.2025.05.009

**Published:** 2025-05-30

**Authors:** Yahya Alwatari, Yazan AlJamal, Suraj M. Yalamuri, Phillip G. Rowse, Dennis A. Wigle

**Affiliations:** Division of Thoracic Surgery, Department of Surgery, and Department of Cardiovascular Surgery, Mayo Clinic, Rochester, Minn

**Figure:**
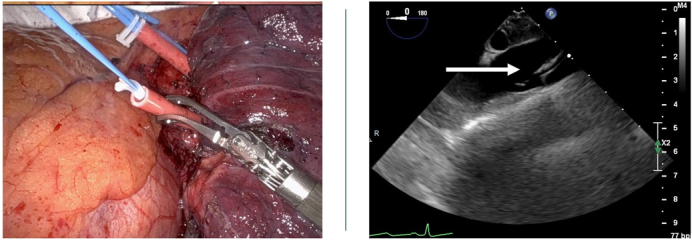
PA control during minimally invasive lung resection.

**Central Message**

Effective control of the PA can potentially minimize bleeding complications during complex lung resection and ensure patient safety.

Although catastrophic bleeding complications during lung resection are rare, left upper lobectomy is associated with the highest overall incidence of life-threatening intraoperative hemorrhage for any individual anatomic lobectomy.^1^ We describe 2 options for pulmonary artery (PA) control: the use of endovascular balloons and Rummel tourniquets. We highlight their respective techniques, benefits, and the scenarios in which they can be used. Institutional Review Board approval was not required; consent was waived.

## Surgical Technique

### PA Endoballoon Occlusion Procedure

**Figure 1:**
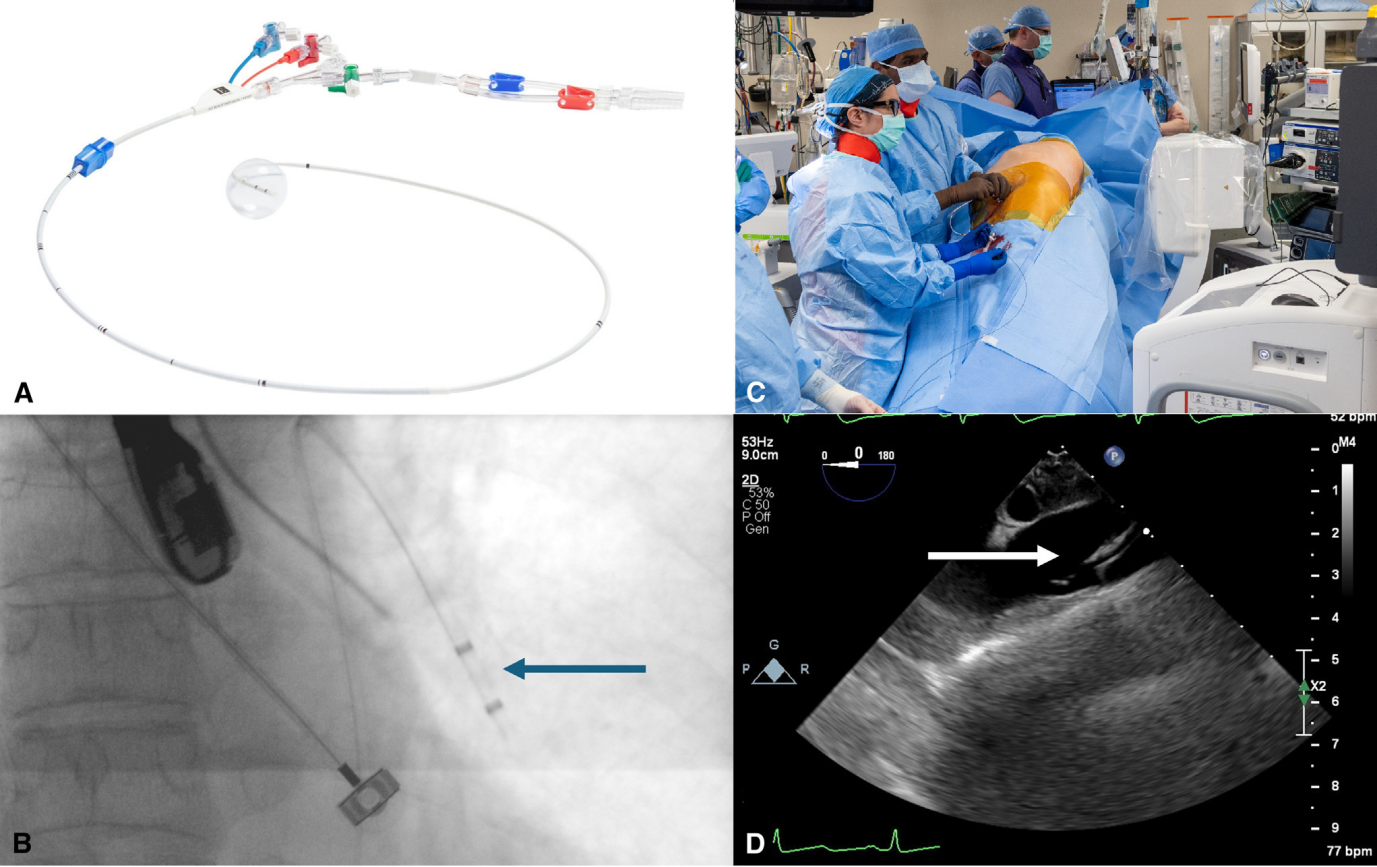
A, IntraClude endoballoon. B-D, Fluoroscopy and TEE confirmation of balloon placement. *Blue arrow* indicates tip of IntraClude endoballoon confirmation using fluoroscopy. *White arrow* indicates tip of IntraClude endoballoon confirmation using echocardiography.

Endovascular control of the PA is achieved via percutaneous placement of the IntraClude endoballoon into the left main PA using both transesophageal echocardiogram (TEE) and C-arm fluoroscopy confirmation (Figure 1). The patient is positioned in a lazy lateral position. Please refer to the Appendix E1 for details on equipment and setup.

1. Vascular Access: Obtain vascular access to the left common femoral vein under ultrasound guidance, followed by the placement of a 16F sheath using the Seldinger technique.
2. Catheter Positioning: Position a 100-cm balloon tip catheter through the sheath into the left main PA with the aid of fluoroscopy. Obtain a PA wedge pressure.
3. Wire Placement: Float a 0.035 Amplatz super stiff wire with a 7-cm floppy tip (260 cm in length) into the PA catheter. Confirm tip position within the distal left main PA using fluoroscopy and TEE.
4. Catheter Removal: Remove the PA catheter, and then position the previously de-aired IntraClude endoballoon over the wire and into the left main PA with the aid of fluoroscopy and TEE.
5. Balloon Inflation: Remove the wire and briefly inflate the balloon to ensure good left main PA flow occlusion with the appropriate wedge pressure. Confirm this with TEE imaging and later with intraoperative inspection and gentle palpation.
6. Achieving Occlusion: Achieve occlusion of the left PA with only 3 to 5 mL of inflation to the balloon, corresponding to a balloon pressure of 80 to 100 mm Hg.
7. Balloon Position Secure: Deflate the balloon and ensure it is well positioned in the left main PA. Anchor it to the skin at the groin with sutures. Proceed with the robotic left upper lobectomy in a standard fashion.

### Intraoperative PA Control With Rummel Tourniquet

**Figure 2:**
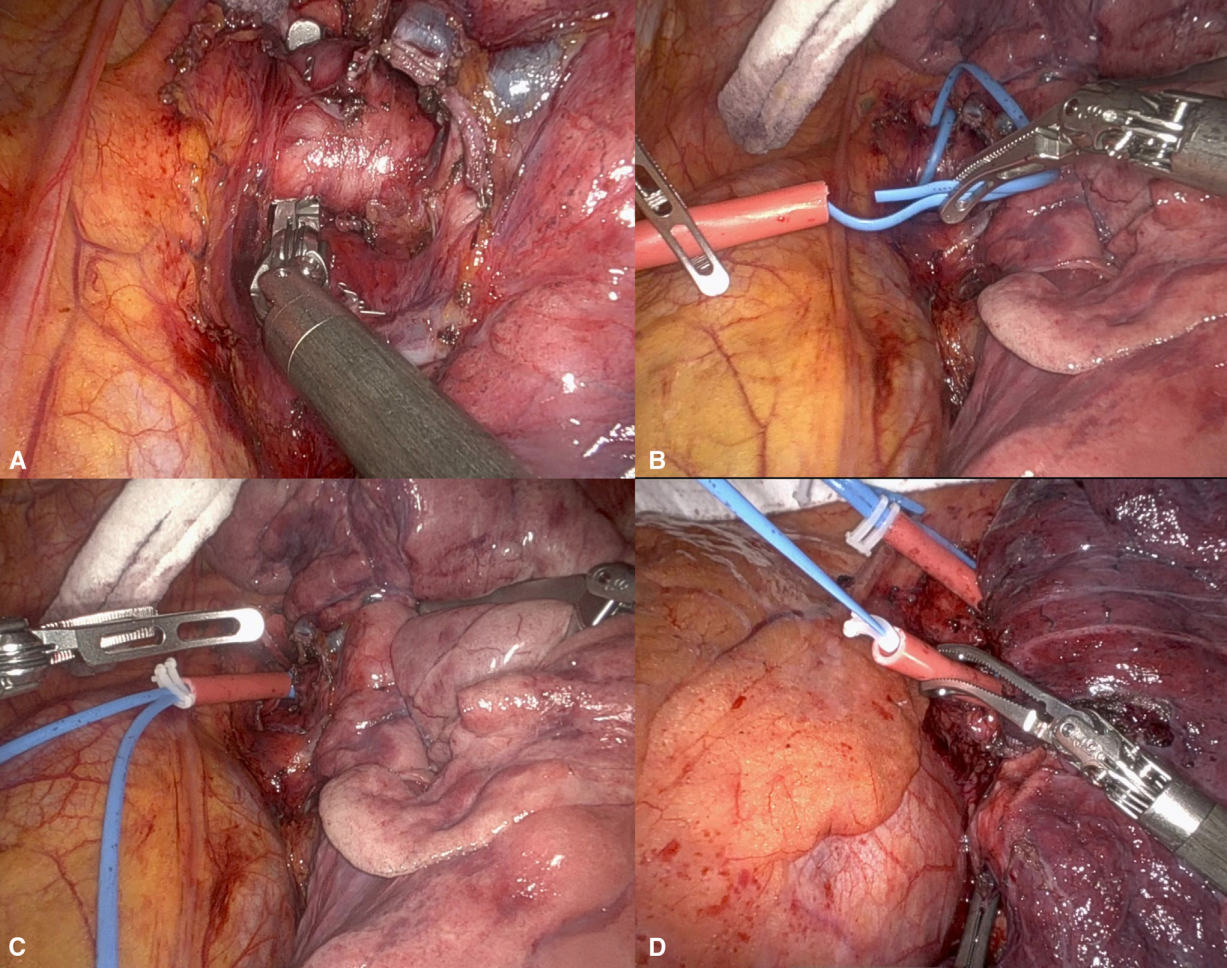
A, Dissection around the left main PA. B, and C, Rummel tourniquet placement. D, Superior PV control with a tourniquet and inferior PV with a bulldog clamp.

In cases when the decision for PA control is made intraoperatively and the patient anatomy and disease process allow proximal access to the proximal left PA, control with a Rummel tourniquet is used (Figure 2).

1. Dissection: Open the mediastinal pleura anteriorly, remove station 5 lymph nodes, and open the plane between the left PA and the aorta. Enter the plane of Leriche, dissecting the proximal left PA circumferentially.
2. Rummel Tourniquet: Use a double-tied rubber vessel loop (30 cm) and a red rubber catheter (3 cm) to create the tourniquet.
3. Securing the Tourniquet: After occluding the PA, use 2 Hem-o-lok clips to secure the Rummel tourniquet. If full pulmonary circulation isolation is required, a similar tourniquet can be applied to the left superior or inferior pulmonary vein. Alternatively, a Bulldog clamp can be used.

## Discussion

Pulmonary circulatory occlusion can be achieved for the right or left lung and can be partial or complete (both PA and pulmonary vein) based on the complexity of the case. Endoballoon use should be planned and performed at the start of the case, whereas the Rummel tourniquet can be used at any time. For cases where dissection around the main PA is not possible due to proximity of disease, frailty of tissue, or radiation changes, then the endoballoon can be a feasible alternative. It is important to remember that cardiopulmonary bypass or venoarterial extracorporeal membrane oxygenation can be used if these techniques cannot achieve proximal PA control.

We have used the left main PA Rummel tourniquet in 2 patients with robotic left upper lobe apical tri-segmentectomy and 1 robotic left upper lobectomy. One of the cases had severe adhesions proximally on the left upper lobe bronchus with the main PA behind it, and 1 case had severely dense lymph nodes around the PA. We used the endoballoon for 3 robotic left upper lobectomies, 1 with metastatic station 5 disease status post–neoadjuvant chemoimmunotherapy. The endoballoon also was used in 2 open cases, 1 with a broncholith invading the right mainstem bronchus requiring sleeve resection and esophagus repair, and 1 with cT3N2 persistent disease after definitive chemoradiation for a squamous cell carcinoma of the left lower lobe and chest wall invasion.

We were able to complete the cases with no perioperative major hemorrhagic events, bleeding requiring blood transfusion, pulmonary thrombosis, or mortality. With the cases we have completed, we have encountered 1 case of balloon dislodgement, which was promptly identified and repositioned. PA control significantly expedited the surgical progress.

## Conclusions

Effective control of the PA can potentially minimize bleeding complications during complex lung resection and ensure patient safety.

## Conflict of Interest Statement

The authors reported no conflicts of interest.

The *Journal* policy requires editors and reviewers to disclose conflicts of interest and to decline handling or reviewing manuscripts for which they may have a conflict of interest. The editors and reviewers of this article have no conflicts of interest.

## Figures and Tables

**Figure E1:**
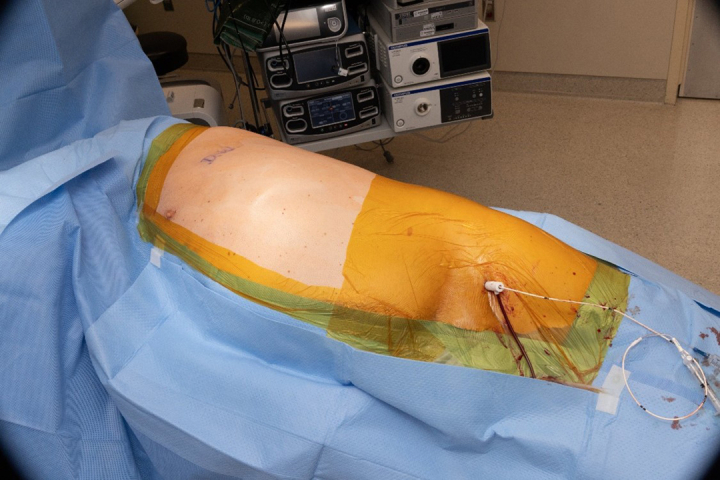
Patient positioning.

**Figure E2:**
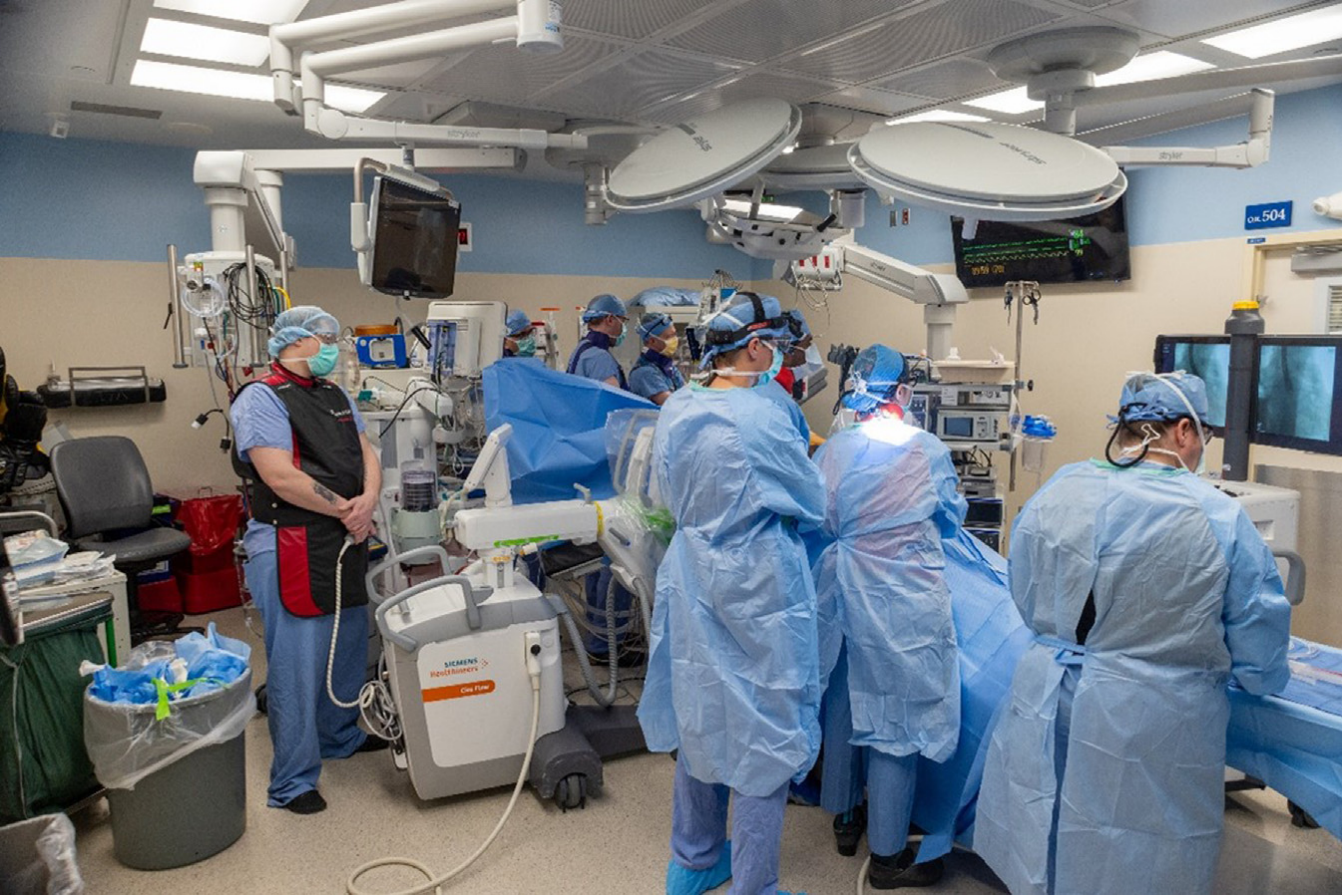
Room setup.

**Figure E3:**
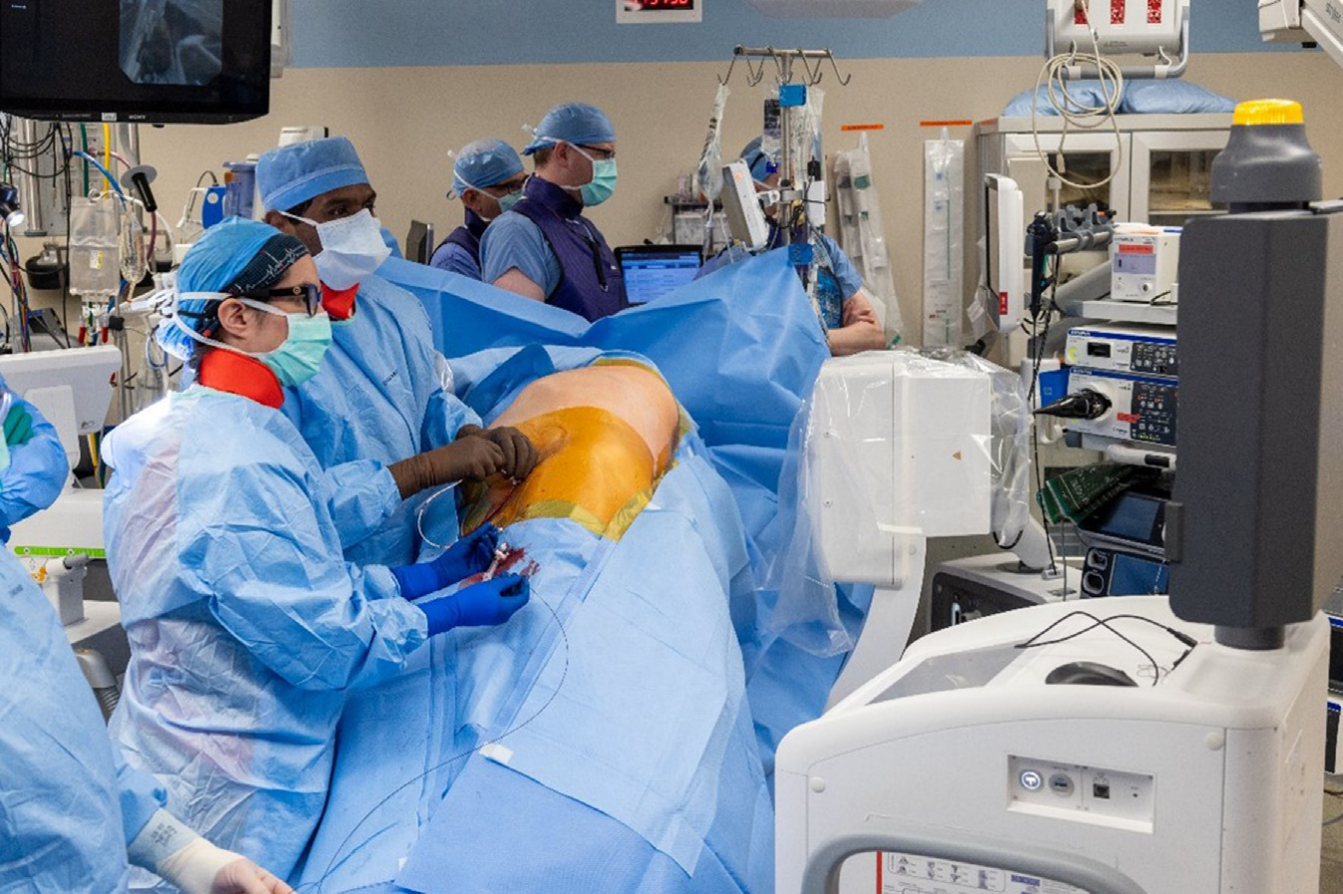
Endoballoon placement.

**Figure E4:**
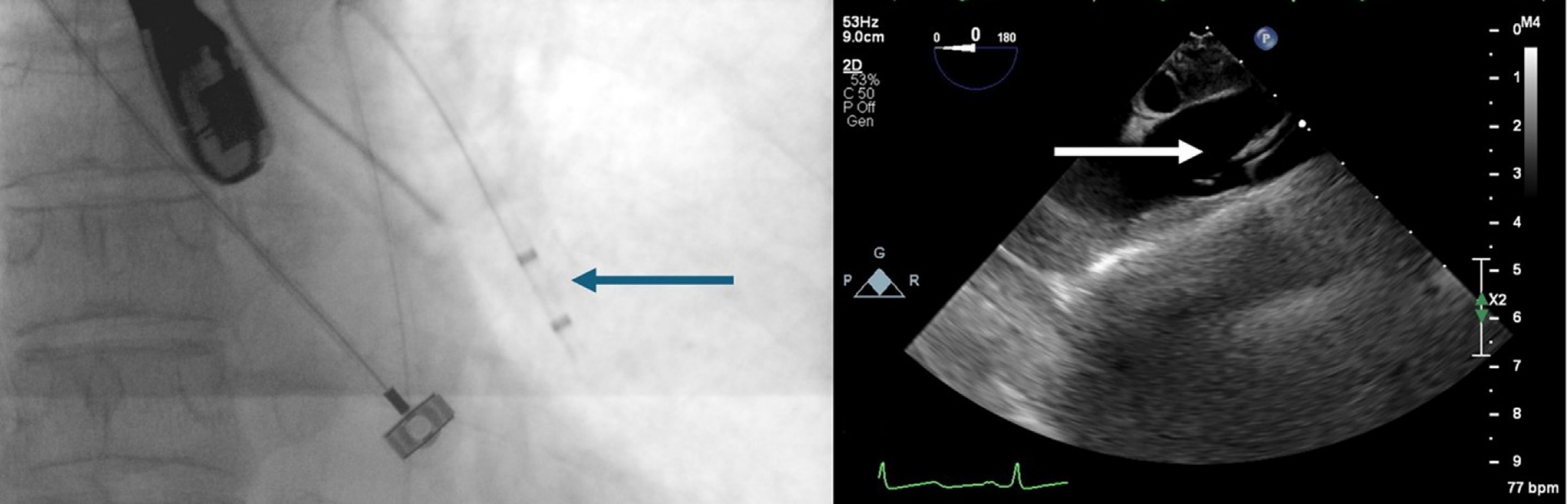
Endoballoon positioning confirmation.

**Figure E5:**
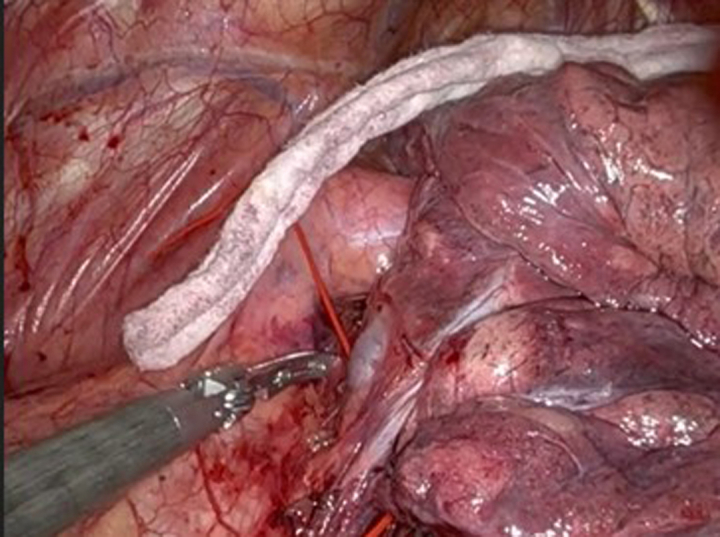
Confirming intraoperatively the endoballoon position.

## Appendix E1. Pulmonary Artery Occlusion Procedure (Position, Equipment List, and Technique)

1. Patient Position/Prep: Underbody Bair Hugger. Neck, torso, and bilateral groins were prepped and draped in sterile fashion. The patient is positioned in a suboptimal (sloppy) lateral position to provide access to the femoral vessels (Figure E1).
2. Equipment List
• 4F Micropuncture kit
• 7F sheath with a sheath wire (pinnacle)
• 12F dilator
• 16F CheckFlo introducer RCF-16.0385
• 7F Balloon wedge (ref #Al-07127)
• 260-cm angled Glidewire
• 260-cm Amplatz SuperStiff with a J tip (floppy 7 cm)
• IntraClude endoballoon (ICF-100)
• V 1.8260-cm wire
• Coda balloon (do not open) 32, 46
• Definity contrast
• Heparinized saline
• C-arm sterile drape
• 2 silk sutures × 2
• Torque
• Cell Saver line × 1/connector for robotic suction
• 6-ft pressure lines × 2
• 11-blade
• Red rubber catheter
• Defib pads
• 12 mL slip-tip/12 mL Leur-Lock syringe connector
3. Equipment Position
• Ultrasound on the patient's left side. Small linear probe.
• C-arm on the patient's right side. Position over the patient's chest. Make sure the emitter for the C-arm is coming through the spine and the receiver plate is on the patient's chest.
• TEE up by anesthesia
• Autotrans above the robot on the right side (Figure E2)
4. Techniques and Steps

Endovascular control of the PA is achieved via percutaneous placement of the IntraClude endoballoon into the left main PA using both TEE and C-arm fluoroscopy confirmation.

1. Vascular Access: Obtain vascular access to the left common femoral vein under ultrasound guidance, followed by the placement of a 16F sheath using the Seldinger technique.
2. Catheter Positioning: Position a 100-cm balloon tip catheter through the 16F sheath into the left main PA with the aid of fluoroscopy via a sterile C-arm. Obtain a PA wedge pressure (Figure E3).
3. Wire Placement: Float a 0.035 Amplatz super stiff wire with a 7-cm floppy tip (260 cm in length) into the PA catheter. Confirm the floppy wire tip within the distal left main PA using both fluoroscopy and TEE.
4. Catheter Removal: Remove the PA catheter over the wire, leaving the wire in the distal left main PA. Position the previously de-aired IntraClude endoballoon over the wire and into the left main PA with the aid of fluoroscopy and TEE guidance (Figure E4).
5. Balloon Inflation: Remove the wire and briefly inflate the balloon with indocyanine green to ensure good left main PA flow occlusion with the appropriate wedge pressure. Confirm this with TEE imaging and later on with intraoperative inspection and gentle palpation. Despite our years of using indocyanine green for intra-aortic balloon confirmation during robotic mitral surgery, we did not find it as successful for PA placement.
6. Achieving Occlusion: Achieve occlusion of the left PA with only 3 to 5 mL of inflation to the balloon, corresponding to a balloon pressure of 80 to 100 mm Hg.
7. Balloon Position Secure: Deflate the balloon and ensure it is well positioned in the left main PA. Anchor it to the skin at the groin with sutures. Proceed with the robotic left upper lobectomy in a standard fashion (Figure E5).

### Complications

Endoballoon aortic occlusion, although useful in cardiac surgery, has been reported to be associated with various complications, including distal embolization, lower-limb ischemia, dissection, and insertion site vascular complications. With the cases we have completed, we have encountered only 1 case of balloon dislodgment, which was promptly identified and repositioned without injury or further complication.

It is important to emphasize that a cardiac surgeon who assisted our thoracic surgery team with the endoballoon placement was always available on standby for all performed surgeries in case cardiopulmonary bypass or venoarterial extracorporeal membrane oxygenation was needed promptly.

## References

[1] Manfredini B., Zirafa C.C., Romano G. (2023). Intraoperative catastrophes during robotic lung resection: a single-center experience and review of the literature. Life (Basel).

